# Supplementary material for: Natural language processing to evaluate texting conversations between patients and healthcare providers during COVID-19 Home-Based Care in Rwanda at scale
Source: PLOS Digit Health. 2025 Jan 15;4(1):e0000625. doi: 10.1371/journal.pdig.0000625 (PMC11734906; doi:10.1371/journal.pdig.0000625)
Supplement: S1 Fig — (PDF) [file pdig.0000625.s001.pdf]

S1 Fig. Defined topics and subtopics of interest were determined by a Delphi process. They were contributed by expert members of the study team and incorporated into a bespoke annotation software used to label conversations.

Topic	Subtopic	Definition and Examples
Symptoms	Physical	Any physical symptom, sensation, or feature (pleasant and unpleasant) that is mentioned in the conversation.<br><br>Examples: pain, weight loss, burning, fatigue, shortness of breath, fever, diarrhea, muscle aches, cramping, coughing, bleeding, broken arm etc.
	Mental / Emotional	Any mental/emotional feeling, sensation, or feature (pleasant and unpleasant) that is mentioned in the conversation.<br><br>Examples: confusion, depression, fear, anxiety, low energy, extreme mood, suicidal ideation, feelings of guilt, lack of interest, stigma etc.
	No Symptoms	Patient explicitly mentions not having symptoms.<br><br>Examples: I have no symptoms, I feel fine.
Diagnostic Methods	Clinical	The process of identifying a disease, condition, or injury based on the signs and symptoms a patient is having and the patient's health history and physical exam.
	Laboratory/Testing	Discussion related to tests on blood, urine, or other tissues or substances taken from the body to help diagnose disease or other conditions, whether performed or not.<br><br>Examples: blood tests (CBC, FBG, Hb1Ac, TSH, lipid panel, etc.), blood cultures, urinalysis, pulmonary function tests, biopsies, stool test (FIT, FOBT), etc.

	Imaging	Studies that provide a picture of the body's interior to help diagnose disease or other conditions.<br><br>Examples: x-ray, CT, MRI, fMRI, ultrasound, PET scan, etc.
	Other	Other diagnostic methods/topic(s) not listed.
Treatment (Rx)	Medications	Pharmaceutical drug that usually requires a medical prescription to be dispensed.<br><br>Examples: antibiotics, antidepressants, anti-hypertensives, amoxicillin, escitalopram, ramipril, etc. include over-the-counter medications (e.g., Tylenol)
	Procedures	Surgery, Breathing procedures, Radiation Therapy etc.
	Alternative	Non-traditional method of healing/treating disease/condition.<br><br>Examples: herbal medicine, acupuncture, massage therapy, naturopathy, meditation, etc.
	Physical Therapy	Care that aims to ease pain and helps function, move, and live better.<br><br>Examples: physiotherapy (geriatric, neurological, orthopedic, chiropractic etc.)
	Counselling	Giving advice/coaching/making recommendations to the patient (can be non-health related).<br><br>Examples: psychologic counseling, coaching of lifestyle modifications like diet beyond standard clinical advice (e.g., in-depth discussion on how to reduce salt intake, not simply saying to reduce salt), smoking cessation, the importance of exercise on cardiovascular health, etc.
	Other	Other treatment topic(s) not listed.

Prevention	Pharmaceutical Prevention	Any mention of an intervention that utilizes chemical or biological methods given to or taken by humans in an attempt to prevent disease.<br><br>Examples: prophylactic medications, immunizations, treatment as prevention, etc.
	Non-Pharmaceutical Prevention	Any mention of an intervention that does NOT rely on bio/chemical products given to or taken by humans in an attempt to prevent disease. Especially those methods that are behavioural (ex: physical distancing) or structural (ex: laws or policies).<br><br>Examples: personal protective equipment (e.g., masking), physical distancing, barriers, quarantine/isolation, lockdowns, school closures, education programs, policies, laws, testing without treatment, environmental decontamination, animal/pest control, etc.
Healthcare Logistics	Outpatient logistics/scheduling	Mention or pertaining to setting up or attending outpatient clinical care or diagnostics.<br><br>Examples: clinic, family doctor office, ECG appointment, hemodialysis appointment, dentist appointment, specialist appointment, physiotherapy, etc.
	Hospitalization	Mention or pertaining to any admission or stay in hospital. Receiving medical treatment during hospital admission.<br><br>Examples: visit to emergency department, or regarding planning, admission, or discharge from hospital care. Includes long-term care facilities.
Lifestyle/Behavioural	Diet / Nutrition	Relating to food/liquid intake and health.

		Examples: nutrition related to weight management, apple, fruits, pie, sandwich, water, Coca-Cola, dinner, lunch, waffles, chips, popcorn, fish, steak, etc.
	Exercise	Relating to physical activity done to improve health and fitness.<br><br>Examples: working out, going to the gym, jogging, playing soccer, playing basketball, doing yoga, walking, walking the dog, etc.
	Substance Use	Relating to the use of drugs or alcohol, and includes substances such as cigarettes, illegal drugs, prescription drugs, inhalants, solvents, etc.<br><br>Examples: alcohol, marijuana, tobacco use, fentanyl, ecstasy, cocaine, addiction of substances etc.
	Other	Other lifestyle topic(s) not listed.
Social/Environmental	Housing	Relating to housing, shelter.<br><br>Examples: “living at home currently,” “bought a new house,” “moving into an apartment in Victoria,” “can’t afford a place,”” “got evicted,” “am homeless”.
	Work/School	Relating to work or school.<br><br>Examples: “studying psychology,” “UBC,” “lots of studying to do,” “stressed from exams,” “I’m a banker,” “work was so busy,” “got fired,” etc.
	Social Services	Relating to government services provided for the benefit of the community, such as education, medical care, and housing programs.

		E.g.: family benefits, welfare, income assistance, housing assistance, food subsidies, foster care, disability support, mental health services, etc.
	Friends & Family	Relating to friends and family relations.<br><br>E.g.: “went to see my mom,” “spent time with friends,” ” hiking with my partner,” “family dinner,” ”hung out with my best friend,”
	Cultural/Religion	Relating to ideas and customs of a culture.<br><br>Examples: “Chinese herbal medicine,” “fasting for Ramadan,” “sweat lodges,” religious and spiritual practices, cultural institutions i.e., church, having own “ideas” about their own illness based on their culture, refusing conventional treatment because of what is taught in their culture.
	Travel	Mention or related to transportation and or travel.<br><br>E.g., vacation in Mexico, work abroad, traveled to… I’m currently in Toronto.
	Physical Environment/Climate	Mention or pertaining to the climate or physical environment.<br><br>E.g., smoke from fires, sun exposure, cold related illness, concerns re logging or farming practices
	Financial	Mention or pertaining to personal or other finances.<br><br>E.g., cost of something, affordability, economics.
	Other	Other social topic(s) not listed.
Service Quality	Problems Solved	An attempt that addresses the patient’s problem within the context of one conversation, regardless of if it was ultimately effective, but at least addressed/attempted to address a present issue

	Grateful Patient	Demonstration of gratitude toward the HCP or care provided.<br><br>Examples: Thanks! (if appears as genuine gratitude, beyond routine customary formalities). I really appreciate. (NOT: See you later, thanks!)
	Service Complaint	Conversation contains any concern or complaint brought forward about the care one is receiving, even if it also contains positive mentions.
	Request to Stop	Select this box if, at any time, the patient requests to be removed from the messaging platform or stop receiving messages.
Safety Concerns	No Subtopics	Discussing worry or concern about their life or the life of others based on specific circumstances.<br><br>Example: I am worried about quarantining in a healthcentre where people are contagious.
Technical/IT	No Subtopics	Non-healthcare technical issues.<br><br>Examples: trouble with texting or health IT, video conferencing and virtual care access
Maternal & Child Health	No Subtopics	Any mention or pertaining to pregnancy, breastfeeding, motherhood, and early childhood care
Health Education	No Subtopics	Providing information to help patients beyond simple advice, with the intent to increase knowledge - can be verbal, written or in the form of visual aids like pamphlets, courses.<br><br>Examples: pamphlet on what diabetes is, teaching how to self-monitor blood pressure, etc.
Stigma	No Subtopics	A mention of the disapproval of, or discrimination against, an individual or group based on perceivable characteristics that serve to distinguish them from other members of a society. Social

		stigmas are commonly related to culture, gender, race, socioeconomic class, age, sexual orientation, body image, intelligence or lack thereof, and health. Can be self-directed.

4
