# Supplementary material for: Natural language processing to evaluate texting conversations between patients and healthcare providers during COVID-19 Home-Based Care in Rwanda at scale
Source: PLOS Digit Health. 2025 Jan 15;4(1):e0000625. doi: 10.1371/journal.pdig.0000625 (PMC11734906; doi:10.1371/journal.pdig.0000625)
Supplement: S2 Fig — (PDF) [file pdig.0000625.s002.pdf]

S2 Fig. NLP Model Details

Traditional machine learning feature extraction details

Feature Extractor	Detail
Character n-gram	Experimented with word bound character ranges of 1-3, 1-4, and 1-5.

Traditional machine learning model hyperparameters and random search ranges

Model	Hyperparameter	Range
**Logistic Regression**	**C** (inverse of regularization strength)	Loguniform (1e-4, 1e4)
	**Penalty**	L1, L2
**Random Forest**	**Max_features** (number of features to consider when looking for best split)	Uniform (0,1)
	**Min_samples_leaf** (minimum number of samples required to be at a leaf node)	Randint (1, 20)
**Ridge Classifier**	**Alpha** (regularization strength)	Loguniform (1e-2, 1e3)

Transformer model hyperparameters

Model	Epochs	Learning Rate	Loss	Optimizer	Batch Size
AfriBERTa[a]	15	Starts at 0.01*	BCEWithLogitsLoss	AdamW	32
AfroXLMR[b]	15				
AfroLM[c]	15				
KinyaBERT[d]	15	10^{-5}			

** We utilized the ReduceLROnPlateau scheduler that reduces the learning rate by a factor of 0.5 for every two epochs where the validation loss does not decrease, up to a minimum value of 10^{-5}.*

[a] https://aclanthology.org/2021.mrl-1.11/

[b] https://arxiv.org/abs/2204.06487

[c] https://arxiv.org/abs/2211.03263

[d] https://huggingface.co/jean-paul/KinyaBERT-small
