# Supplementary material for: Natural language processing to evaluate texting conversations between patients and healthcare providers during COVID-19 Home-Based Care in Rwanda at scale
Source: PLOS Digit Health. 2025 Jan 15;4(1):e0000625. doi: 10.1371/journal.pdig.0000625 (PMC11734906; doi:10.1371/journal.pdig.0000625)
Supplement: S3 Fig — (PDF) [file pdig.0000625.s003.pdf]

S3 Fig. Frequency and proportion of conversations in which all topics and subtopics of interest occurred within the human-labelled, machine-labelled, and complete corpora. Note: Conversations were often labelled with multiple topics/subtopics, therefore percentages do not sum to 100%.

	Human Labelled (n=2,791)	Machine Labelled (n=9,328)	Human-and-Machine-Labelled (n=12,119)
**Topic** Subtopic(s)	n (%)	n (%)	n (%)
**Symptoms**	**2,002 (72)**	**6,305 (68)**	**8,307 (69)**
None	1,657 (59)	-	-
Physical	406 (15)	-	-
Mental/Emotional	45 (1·6)	-	-
**Diagnostic Methods**	**1,030 (37)**	**3,539 (38)**	**4,569 (38)**
Laboratory/Testing	1,018 (36)	-	-
Imaging	7 (0·2)	-	-
Clinical	5 (0·2)	-	-
Other	5 (0·2)	-	-
**Prevention**	**459 (16)**	**1,685 (18)**	**2,114 (18)**
Pharmaceutical	17 (0·6)	-	-
Non-pharmaceutical	443 (16)	-	-
**Service Quality**	**425 (15)**	**NP**	**NP**
Grateful Patient	244 (8·7)	-	-

Service Complaint	106 (3·8)	-	-
Request to Stop	50 (1·8)	-	-
Problems Solved	45 (1·6)	-	-
**Social**	**406 (15)**	**1,896 (20)**	**2,302 (19)**
Cultural/Religion	154 (5·5)	-	-
Friends & Family	138 (4·9)	-	-
Financial	53 (1·9)	-	-
Work/School	46 (1·7)	-	-
Housing	32 (1·2)	-	-
Social Services	30 (1·1)	-	-
Travel	12 (0·4)	-	-
Environment/Climate	6 (0·2)	-	-
Other	7 (0·3)	-	-
**Healthcare Logistics**	**281 (10)**	**1,599 (17)**	**1,880 (16)**
Logistics/Scheduling	268 (9·6)	-	-
Hospitalization	15 (0·5)	-	-
**Technical/IT**	**185 (6·6)**	**NP**	**NP**
**Treatment**	**174 (6·2)**	**791 (8·5)**	**965 (8·0)**
Medications	152 (5·5)	-	-
Alternative	35 (1·2)	-	-
Physical Therapy	1 (0·04)	-	-

Procedures	0 (0)	-	-
Counseling	0 (0)	-	-
Other	0 (0)	-	-
**Lifestyle/Behavioural**	**156 (5·5)**	**NP**	**NP**
Diet/Nutrition	102 (3·7)	-	-
Exercise	25 (0·9)	-	-
Substance Use	0 (0)	-	-
Other	52 (1·9)	-	-
**Health Education**	**45 (1·6)**	**NT**	**NT**
**Maternal & Child Health**	**33 (1·2)**	**NT**	**NT**
**Safety Concern**	**20 (0·7)**	**NT**	**NT**
**Stigma**	**15 (0·5)**	**NT**	**NT**

NP = Not Predictable (best model did not meet F1 performance cutoff and was not applied)

NT = Not Tested (insufficient data to develop models)
