# Supplementary material for: Natural language processing to evaluate texting conversations between patients and healthcare providers during COVID-19 Home-Based Care in Rwanda at scale
Source: PLOS Digit Health. 2025 Jan 15;4(1):e0000625. doi: 10.1371/journal.pdig.0000625 (PMC11734906; doi:10.1371/journal.pdig.0000625)
Supplement: S1 Table — (PDF) [file pdig.0000625.s004.pdf]

S1 Table. Comparison of the odds of discussion of predictable conversation topics of interest (i.e., those conversation topics of interest for which we developed a classification model of interest meeting the F1≥0.7 performance cutoff) by patient demographic and clinical factors in the complete conversation corpus (n=12,119). Presented p-values were adjusted for multiple testing using the Benjamini-Hochberg correction.

TOPIC	COMPARISON (vs. reference)	ODDS RATIO (95% CI, adj. p-value)
Symptoms	Female vs. Male	1.04 (0.96-1.13, p=0.39)
	Age (per year increase)	1.00 (1.00-1.00, p=0.47)
	Contact vs. Case	1.25 (1.06-1.47, p=0.02)
Diagnostic Methods	Female vs. Male	0.87 (0.80-0.94, p=0.0012)
	Age (per year increase)	1.00 (0.99-1.00, p=0.060)
	Contact vs. Case	0.84 (0.73-1.00, p=0.060)
Social	Female vs. Male	1.04 (0.94-1.14, p=0.45)
	Age (per year increase)	1.005 (1.002-1.009, p=0.012)
	Contact vs. Case	1.09 (0.91-1.30, p=0.43)
Prevention	Female vs. Male	0.93 (0.84-1.02, p=0.29)
	Age (per year increase)	1.00 (0.99-1.00, p=0.22)
	Contact vs. Case	0.61 (0.49-0.75, p=0.00004)
Healthcare Logistics	Female vs. Male	0.88 (0.79-0.98, p=0.025)

	Age (per year increase)	1.005 (1.0009-1.008, p=0.025)
	Contact vs. Case	0.69 (0.55-0.86, p=0.003)
Treatment	Female vs. Male	1.077 (0.94,1.23, p=0.36)
	Age (per year increase)	1.006 (1.0009-1.011, p=0.048)
	Contact vs. Case	0.30 (0.20-0.46, p<0.0001)

6
