# Supplementary material for: Natural language processing to evaluate texting conversations between patients and healthcare providers during COVID-19 Home-Based Care in Rwanda at scale
Source: PLOS Digit Health. 2025 Jan 15;4(1):e0000625. doi: 10.1371/journal.pdig.0000625 (PMC11734906; doi:10.1371/journal.pdig.0000625)
Supplement: Attachment — Submitted filename: Response to Reviewers_PLOS DH NLP.pdf [file pdig.0000625.s005.pdf]

Response to Reviewers PLOS Digital Health Rwanda COVID NLP Oct 2024

Response to Reviews:

Reviewer #1: This is an interesting, well-written paper. I think you bring up some important issues that will spark considerable debate, both in terms of what you have actually found and in terms of the policy implications. I think an interesting, but unexplored point is whether academics have changed their research strategy in light of the increased expectations of research production. One strategy in particular, using more co-authors is a way in which researchers can increase their productivity, even if space in top journals is limited. This strategy may be particularly salient to macro researchers, given the extremely limited space available.

Our reply: Thank you for your interest and positive comments on the interest, writing, and potential for prompting policy debate. The point about academics changing their research strategy is interesting. We agree there is a shift in the academic landscape and context in line with the San Francisco Declaration on Research Assessment (DORA). Since this topic is tangential to the core focus of our manuscript, we have not included a discussion on how collaboration via co-authorship may influence research productivity, particularly within the realm of health-related digital research in this paper directly, but feel it is worthy topic of a separate article and we would welcome the reviewer to reach out to us on this.

Reviewer #2: This manuscript thoroughly addresses the issue of using natural language processing (NLP) to analyze text conversations between patients and healthcare providers during the COVID-19 pandemic. This paper shows how digital technologies, such as SMS, can be used to support isolated patients on a large scale and in conditions of health restrictions. Different machine learning models are thoroughly evaluated and a comprehensive comparison between the performance of the models in different languages is presented. The findings of the article emphasize that patient support is not limited to medical issues and social issues are also important. By providing solutions to improve health responses in future crises, this research has a high value for public health decisions.

Our reply: Thank you for your positive comments on our findings and methodologies comparing the models in different languages, that social as well as medical issues were discovered, and the high value for public health decision-making.

Reviewer #3: • Usage of NLP within the context of healthcare services seems novel and interesting, but I couldn’t grasp whats the importance of conducting such study and how its findings can help the beneficiaries.

Our replies: Thank you for your comments and detailed points. We have made some changes in the writing, particularly of the Introduction and Discussion to better clarify the importance of this study for readers. Please see point-by-point replies below:

•	Has the manuscript adhered to the journal`s guidelines in terms of format and sequence of sections? If it hasn't adhered, I suggest the authors to revise the manuscript in accordance.

Our Reply: Thank you. We recognize that the order of headings in our original submission included the Results before the Methods, which is allowable but non-standard. The PLOS author section states: "Uniformity in format facilitates the experience of readers and users of the journal. To provide flexibility, however, authors are also able to include the Materials and Methods section before the Results section or before the Discussion section. Please also note that the Results and Discussion can be combined into one Results/Discussion section". We made this adjustment after reviewing a published article in the journal in the current format. However, for improved consistency we have reordered our submission to placing the more standard Methods section prior to the Results section.

•	while the introduction is well-structured and informative, it could benefit from a more detailed discussion on the limitations of previous studies to highlight the novelty of their approach.

Our reply: Thank you, we have improved some wording in the introduction to make the specific research problem (knowledge gap) and question we are addressing clearer.

•	Discuss the previous studies within the literature and the novelty of this study at the end of the introduction section.

Our reply: Thank you. We had included reporting and referencing related studies in the third paragraph of the Discussion (Lines 377-395). Specifically, we provided references of studies that did topic classification of patient-healthcare provider communications and highlighted that they were narrower in scope. However, we have added discussion and references to this context to the Introduction section as well (Lines 104-109, references 12-16.

•	Did u use any sampling technique for selection of the study sample?

Our reply: The training data was a continuous sample from the first phases of the pandemic to start on language translation and prepare data for annotation. This was clarified with improved language in the Methods section and limitations of this approach are discussed in the Discussion.

•	it would be beneficial to include more information on the selection criteria for text messages analyzed and how potential biases were mitigated, particularly regarding language translation challenges with Kinyarwanda texts.

Our reply. Thank you. We have expanded both the methods description of the sampling approach and our discussion of translation related limitations in the Methods and Discussion sections.

•	Does the study have any ethical approval from the corresponding ethical committee?

Our reply: Our apologies, the ethics statement had been removed from the submitted manuscript as the submitting author thought it was a separate section of the PLOS online submission procedure. The statement has been added back (it was approved by both the University of British Columbia and the Rwanda Biomedical Center institutional review boards).

• What is the significance of the findings of this study? who can use such findings? Are these findings important for the beneficiaries?

Our reply: The significance of the study is multiple. First, it investigates and compares the use of various NLP topic classification model performance on real-world public health text conversational data, with findings that have not been published elsewhere. Second, it demonstrates both potential and limitations of current (at the time) NLP models in classifying a low-resource language (e.g. Kinyarwanda). Ultimately this study provides an example of how real-world public health conversational data with monitored patients could be analyzed using NLP models to provide insights into the relative medical and social issues that isolated patients may experience which could ultimately guide outbreak and pandemic response decision-making. It thus illustrates how building on these approaches could assist in future responses. Publishing the application of NLP and AI methods on real-world health data helps iterate between 'theoretical' uses of NLP at scale in these responses toward actualized examples. Stakeholders who can use these approaches or findings include healthcare data scientists working in NLP, and healthcare providers and public health decision makers and advocacy groups (since this approach turns patients' words into actionable data).

• The paper lacks discussion of results with the literature within the context. Elaboration on how these results compare with existing literature could strengthen their significance.

Our reply: The third paragraph of the Discussion (Lines 377-395) places our current study in the context of other published research. We conducted an extensive search of the literature using PubMed and Google Scholar and found few studies that used text classification or other NLP methods to evaluate text conversations between healthcare providers and patients for public health or clinical monitoring. The few studies we found are discussed and referenced. Lines 395-410 contain additional references to place our research in the context of machine learning and translation models with low-resource languages.

• In summary, the methodology of the study and usage of NLP within such context seems interesting; Anyhow, I believe there is no clear rationale behind conduction of such study and I couldn't grasp the research question fully. I suggest the authors to use the same methodology and utilize the same data once again, but this time with a clear research question which seems valuable for readers and beneficiaries. As an example, I believe the data within the text messages could be utilized to obtain an analysis on the elements and principles of patient-centered care within the study population; something which seems to be valuable for the beneficiaries within the context. OR analysis of the views of users of such SMS services provided within their text messages. OR an analysis on the challenges of service users during their utilization of SMS services. OR an analysis on the types of patients who had positive and negative experiences during usage of such SMS services which can provide significant

implications for the beneficiaries. OR any other topic the authors believe as having the potential to provide valuable insights for the beneficiaries. With kind regards.

Our reply: Thank you for this important comment. We have made substantial efforts to improve the wording of the manuscript in our resubmission to more clearly lay out the rationale, research question, findings in relation to previous studies, and importance of the findings and approach. We chose to focus the paper on experimental testing and optimization of NLP classification models and to present the applied findings more descriptively since these findings illustrate the type of information that can be useful to inform public healthcare providers and policy makers. We chose not to pre-formulate further research questions from the data, as this reviewer recommends, on behalf of such stakeholders as we feel this would influence or limit the ways in which the descriptive data could be used by those stakeholders. We agree that using NLP, especially natural language understanding (NLU) and natural language generation (NLG) or generative AI would be a natural next step in helping stakeholders in decision-making but feel this is outside the scope of the current study. We have included this important insight into the Discussion. The three other reviewers reported finding the current study findings would be interesting to readers and stakeholders in its current form.

Reviewer #4: This study provides valuable insights into using SMS-based health monitoring for isolated patients during the SARS-CoV-2 pandemic in Rwanda. The large-scale analysis of patient conversations using NLP, especially in under-resourced languages like Kinyarwanda, is a significant contribution. The comparison of machine learning models further enhances the relevance of your findings for digital health. Your work demonstrates the scalability of remote support in pandemics and offers important insights for future public health responses.

Our Reply: Thank you for your positive feedback.

All edits are provided in tracked changes on the revised manuscript. In addition to the reviews here, we were requested by MedRxIV to change Table 3 to include only English language. Those changes are also reflected as tracked changes in the uploaded revised document.

We thank all of the reviewers for their insightful comments.

Richard Lester,

On behalf of all co-authors and contributors.
